# An update to the Monro–Kellie doctrine to reflect tissue compliance after severe ischemic and hemorrhagic stroke

**DOI:** 10.1038/s41598-020-78880-4

**Published:** 2020-12-16

**Authors:** Anna C. J. Kalisvaart, Cassandra M. Wilkinson, Sherry Gu, Tiffany F. C. Kung, Jerome Yager, Ian R. Winship, Frank K. H. van Landeghem, Frederick Colbourne

**Affiliations:** 1 Department of Psychology, Faculty of Science, University of Alberta, Edmonton, AB Canada; 2 Neuroscience and Mental Health Institute, University of Alberta, Edmonton, Canada; 3 Department of Pediatrics, Faculty of Medicine and Dentistry, University of Alberta, Edmonton, Canada; 4 Department of Psychiatry, Faculty of Medicine and Dentistry, University of Alberta, Edmonton, Canada; 5 Department of Laboratory Medicine and Pathology, University of Alberta Hospital, Edmonton, Canada

**Keywords:** Stroke, Cell death in the nervous system

## Abstract

High intracranial pressure (ICP) can impede cerebral blood flow resulting in secondary injury or death following severe stroke. Compensatory mechanisms include reduced cerebral blood and cerebrospinal fluid volumes, but these often fail to prevent raised ICP. Serendipitous observations in intracerebral hemorrhage (ICH) suggest that neurons far removed from a hematoma may shrink as an ICP compliance mechanism. Here, we sought to critically test this observation. We tracked the timing of distal tissue shrinkage (e.g. CA1) after collagenase-induced striatal ICH in rat; cell volume and density alterations (42% volume reduction, 34% density increase; *p* < 0.0001) were highest day one post-stroke, and rebounded over a week across brain regions. Similar effects were seen in the filament model of middle cerebral artery occlusion (22% volume reduction, 22% density increase; *p* ≤ 0.007), but not with the Vannucci-Rice model of hypoxic-ischemic encephalopathy (2.5% volume increase, 14% density increase; *p* ≥ 0.05). Concerningly, this ‘tissue compliance’ appears to cause sub-lethal damage, as revealed by electron microscopy after ICH. Our data challenge the long-held assumption that ‘healthy’ brain tissue outside the injured area maintains its volume. Given the magnitude of these effects, we posit that ‘tissue compliance’ is an important mechanism invoked after severe strokes.

## Introduction

Elevated intracranial pressure (ICP) constitutes a serious life-threatening emergency. Following brain injury, ICP rises, often due to space-occupying mass effects of intracranial bleeds and/or edema. Edema and electrolyte imbalances impair brain function, and high ICP can initiate a vicious cycle worsening edema and injury^1,2^, or even cause death via brainstem transforaminal herniation^3,4^. Several compliance mechanisms blunt these ICP increases; notably, cerebrospinal fluid (CSF) and intravascular blood are redirected out of the cranium to create space^1,5,6^. Given the relatively small volume of blood and CSF within the cranium, and their necessity, it is not surprising that these compliance mechanisms are overwhelmed, especially after severe injury or stroke^3,5,7^.

According to traditional pressure–volume relationships outlined in 1783 by Monro and Kellie^1,8^, as ICP increases, vascular blood and CSF are displaced as part of a dynamic equilibrium to help maintain normal pressure within the inelastic cranium, while brain tissue remains unchanged. In the past few decades, it became clear that neurons are not as immutable as once thought; indeed, neurons are able to individually and quickly change their volume^9–11^, especially in response to potential injury via osmotic or mechanical stress (e.g. elevated ICP)^12,13^. The brain parenchyma makes up ~ 80% of intracranial contents, with 50–60% of that consisting of grey matter^14^. If tissue compliance freed up 5% of the volume occupied by gray matter in response to elevated ICP, that would represent a savings of 65 mL within the human cranium, (~ 1200–1600 mL)^15^. Given that the average hematoma size is ~ 35 mL^15^, tissue compliance could substantially offset the mass effect caused by bleeds and/or cerebral edema.

Although the existence of adaptive neuronal volume regulation has been recognized for some time, the capacity for neurons to participate in pressure compliance dynamics has never been explored in vivo after injury. Recently, we observed that after large striatal ICH (collagenase and autologous whole blood (AWB) models), neurons in distal brain regions, such as hippocampus and cortex, decrease in size and pack closer together. We postulated that this is a compliance mechanism to counter high ICP^16^. These conclusions arose from serendipitous observations, without an a priori hypothesis about tissue compliance. To be certain that our original observations were not a result of chance or HARKing (hypothesizing after results are known), a widespread issue which inflates the false discovery rate^17,18^, we presently sought to replicate and expand upon those preliminary findings. This is part of adopting a “two-pronged approach of exploration-confirmation” as recently recommended to shift the bias against confirmatory research and combat the replication crisis within biomedical research^19^.

Our initial work examined two brain regions at a single survival time using limited methodology, so our purpose here was to elucidate the timing and extent of tissue compliance in ICH using stereology, the gold standard of cellular morphology assessment. First, we stereologically assessed neurons across several regions 1, 3, and 7 days following a large ICH in adult male rats, an injury which results in considerable edema^20,21^, and heightened ICP^22–24^. As we hypothesized that tissue shrinkage is an ICP compliance mechanism, we expected to see the largest effect early after ICH, when ICP is increased^23,25,26^, versus 1 week after ICH, when ICP is reduced, edema has resolved, and hematoma resolution has begun^16,22,26^. Our previous study did not find evidence of cell death, but did not formally test for this possibility^16^. Thus, Fluoro-jade C stain (FJC) was presently used to test whether tissue compliance is associated with cell death.

To establish whether the tissue compliance phenomenon is unique to hemorrhagic stroke, or whether it occurs in other brain injuries that elevate ICP, we also assessed cell morphology following ischemic stroke and hypoxic-ischemic encephalopathy (HIE; Table 1). Our purpose was not to directly compare the extent of shrinkage among brain injury models, but to observe whether it occurred in multiple settings (e.g. whether a hematoma was required). In experiment 2, we used the intraluminal suture occlusion model of middle cerebral artery occlusion (MCAO), a model with considerable edema and elevated ICP^25,27^. In experiment 3, we used a rat pup model of HIE, an insult that does not typically raise ICP^28^. Therefore, we expected to observe tissue compliance changes after MCAO, but to a lesser extent or not at all after HIE. Lastly, we hypothesized that these volume reductions could have detrimental effects on cellular ultrastructure, as cell volume is closely tied to the optimal function of enzymes and other cellular processes^10,12,13^. Thus, in experiment 4, we used transmission electron microscopy (TEM) to image several representative brain areas 24 h after ICH to appraise subcellular perturbations that may result from tissue compliance.

**Table. 1:** Timelines for Experiments 1–4. A total of 82 rats were used in this study.

Experiments in the present study	Experimental groups by post-stroke survival time	Assessments used in each experiment	Brain regions assessed
Experiment 1: ICH time course	ICH-D1 (n = 8)	Cortical thickness	Layer CA1 of the hippocampus (CA1)
	ICH-D3 (n = 8)	Lesion volume	Layer CA3 of the hippocampus (CA3)
	ICH-D7 (n = 8)	Stereological assessment	Primary somatosensory cortex (S1)
	SHAM (n = 12)	Fluorojade C + Cell counts	Contralateral Dorsal Striatum(Striatum)
Experiment 2: MCAO 24 h survival	MCAO-D1 (n = 9)	Cortical thickness	Layer CA1 of the hippocampus (CA1)
	SHAM (n = 8)	Lesion volume	Primary somatosensory cortex (S1)
		Stereological assessment	Contralateral Dorsal Striatum (Striatum)
Experiment 3: HIE 24 h survival	HIE-D1 (n = 10)	Cortical thickness	Layer CA1 of the hippocampus (CA1)
	SHAM (n = 10)	Lesion volume	Primary somatosensory cortex (S1)
		Stereological assessment	
Experiment 4: electron microscopy	ICH-TEM (n = 5)	Qualitative assessment	Layer CA1 of the hippocampus (CA1)
	SHAM (n = 5)		Primary somatosensory cortex (S1)

## Results

### Exclusions and mortality

There was no mortality in experiments 1 and 3. There was one spontaneous mortality of unknown cause in experiment 2 (MCAO-D1 rat). In experiment 1, three rats in the SHAM group, one rat in the ICH-D1 group, one rat in the ICH-D3 group, and two rats in the ICH-D7 group were excluded from all analyses to preserve stereological accuracy.

Based upon a priori tissue quality exclusion criteria, in experiment 1, four rats from the SHAM group, two rats in the ICH-D1 group, and one rat in the ICH-D3 group were excluded from regional S1 analysis. Two SHAM rats, and one ICH-D3 rat were excluded from CA1 analysis. Two SHAM rats and one ICH-D3 rat were excluded from striatum analysis. In experiment 3, one rat in the HIE-D1 group was excluded from lesion volume analysis. These regional analyses exclusions are in addition to total experiment exclusions, and were done in order to avoid bias from histological artefact.

### Experiment 1 (ICH) results

#### Maximum hematoma area

**Figure 1:**
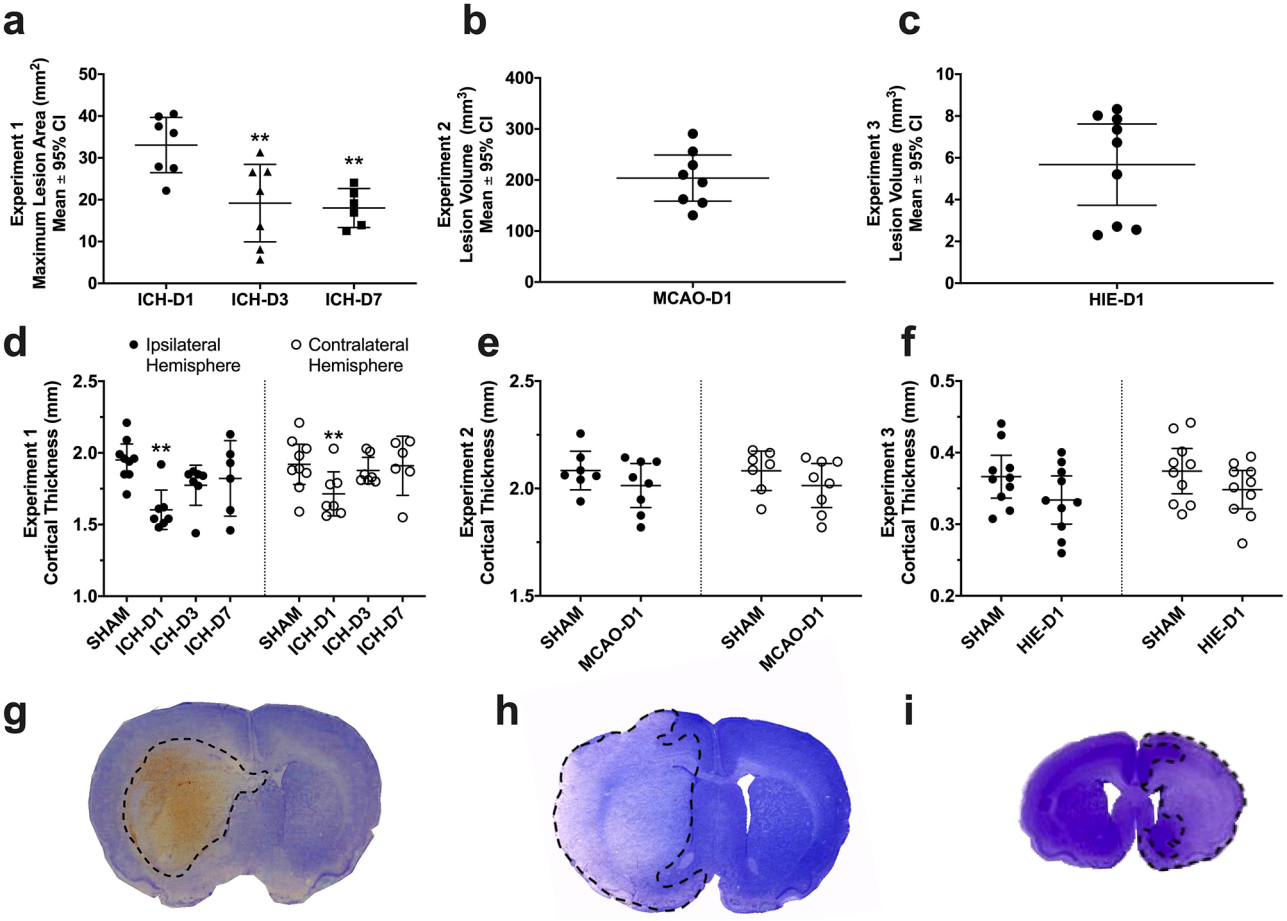
Scatter plots of animal lesion size and cortical thickness in experiments 1 (**a**, **d**), 2 (**b**, **e**), and 3 (**c**, **f**) with representative sections of ICH (collagenase model; **g**), MCAO (intraluminal suture occlusion model; **h**) and HIE (Vannucci–Rice model; **i**). Maximum hematoma area was quantified in experiment 1 (**a**), whereas total lesion volume in was quantified in experiments 2 (**b**) and 3 (**c**). SHAM animal lesion volume data were not added as there were no signs of injury in any of the sham operated rats in these experiments. Cortical thickness (**d**–**f**) was measured from the cingulum to layer II of the cortex bilaterally in each animal, **p < 0.01 versus respective SHAM controls. Scatterplots were made using GraphPad Prism version 8.4.3 for macOS, GraphPad Software, San Diego, California USA, www.graphpad.com.

The maximum hematoma area (Fig. 1a) in the ICH-D1, D3, and D7 experimental groups was 33.1 ± 6.6 μm^2^, 19.2 ± 9.3 μm^2^, and 18.0 ± 4.7 μm^2^, respectively. Injury was largely confined to the striatum (mean ± 95% C.I.). As expected, no signs of injury were seen in SHAM-operated rats.

#### Cortical thickness

The ICH-D1 rats had thinner cortices (1.66 ± 0.07 mm) than SHAMs (1.94 ± 0.11 mm*; p* < 0.001, Cohen’s d = 1.7; Fig. 1d) whereas cortical thickness was similar among ICH-D3 (1.83 ± 0.06 mm) and ICH-D7 (1.87 ± 0.22 mm) compared to SHAM groups (all *p ≥ *0.152). Thus, the cortex was transiently compressed following ICH.

#### Neuron cell volume (S1, CA1, CA3, striatum)

**Figure 2:**
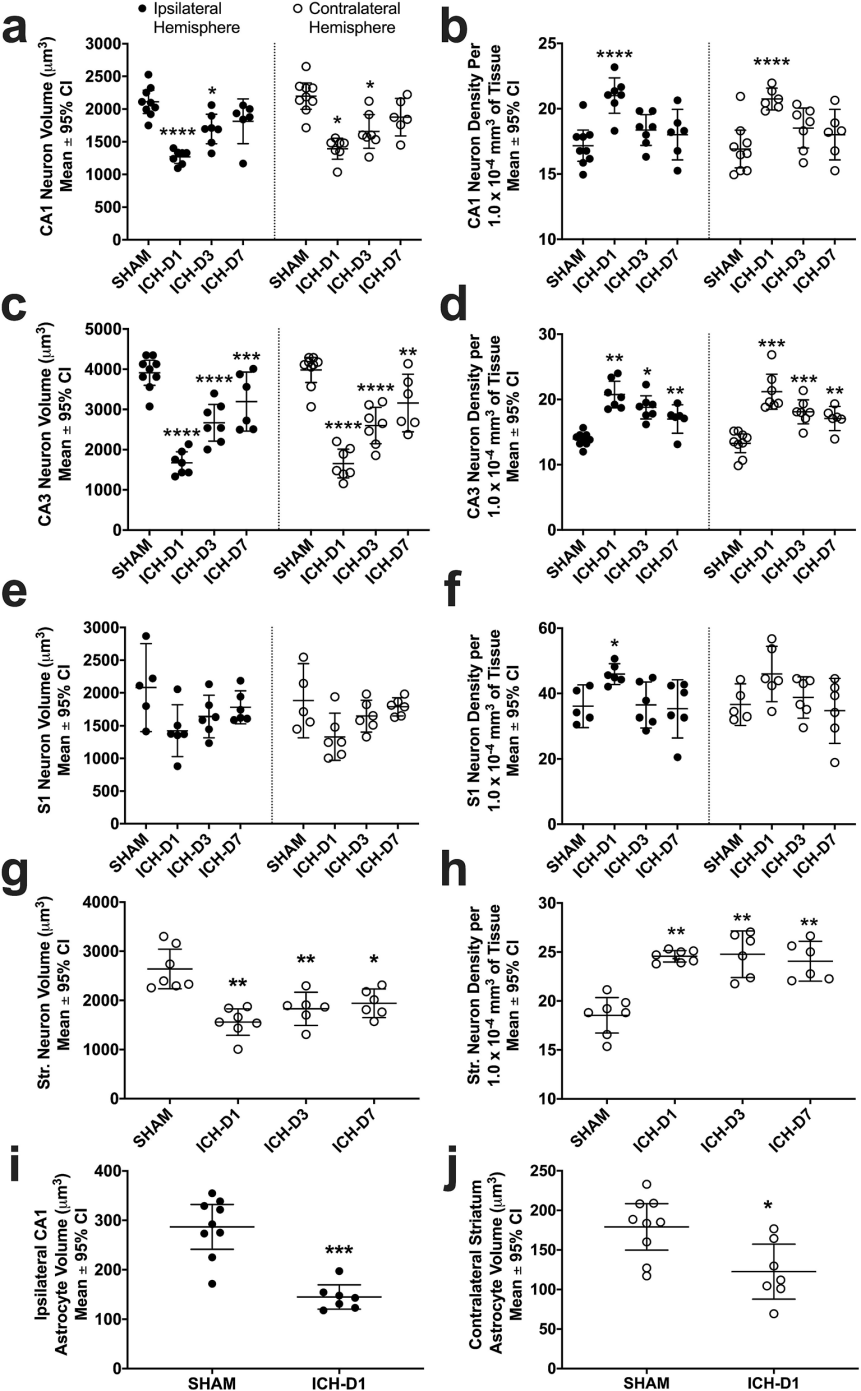
In experiment 1, neuron soma volume (**a**, **c**, **e**, **g**) and density (**b**, **d**, **f**, **h**) were analyzed with unbiased stereology on days 1, 3, and 7 following striatal ICH or sham operation. Astrocyte soma volume was also analyzed on day 1. Areas assessed in all animals included in CA1 (**a**, **b**, **i**), CA3 (**c**, **d**), S1 (**e**, **f**), and contralateral striatum (**g**, **h**, **j**). Ipsilateral striatum was not assessed as it was largely destroyed by the ICH, as expected. *p < 0.05, **p < 0.01, ***p < 0.001, ****p < 0.0001 versus respective SHAM controls. Scatterplots were made using GraphPad Prism version 8.4.3 for macOS, GraphPad Software, San Diego, California USA, www.graphpad.com.

CA1 cell volume was significantly reduced on D1 (1331 ± 121 μm^3^) and D3 (1676 ± 241 μm^3^) compared to SHAMs (2155 ± 189 μm^3^; *p ≤ *0.020, Cohen’s d = 1.8 on D1, and 1.4 on D3). CA3 cell volume was also significantly reduced on D1 (1662 ± 316 μm^3^) and D3 (2633 ± 454 μm^3^) compared to SHAMs (3946 ± 313 μm^3^; *p ≤ *0.013, Cohen’s d = 1.9 on D1, 1.7 on D3). Lastly, contralateral dorsal striatum also had significant cell volume reductions on D1 (1558 ± 269 μm^3^), D3 (1829 ± 338 μm^3^), and D7 (1829 ± 291 μm^3^) compared to SHAMs (2639 ± 403 μm^3^; *p ≤ *0.016, Cohen’s d = 1.4–1.7). Therefore, the ICH appears to have caused widespread cell volume reductions that gradually recovered (Fig. 2).

#### Astrocyte cell volume (CA1, striatum)

Astrocytes in ipsilateral CA1 were significantly smaller in ICH-D1 rats compared to SHAMs (144 ± 24 μm^3^ versus 286 ± 53 μm^3^; *p* < 0.001, Cohen’s d = 3.0). Astrocytes in contralateral striatum were also significantly smaller in ICH-D1 rats compared to SHAMs (123 ± 35 μm^3^ versus 179 ± 30 μm^3^; *p* = 0.0108, Cohen’s d = 1.4). Therefore, astrocytes appear to participate in the tissue compliance response (Fig. 2i–j).

#### Neuron cell density (S1, CA1, CA3, striatum)

Cell density was significantly increased bilaterally on D1 after ICH compared to SHAMs in areas CA1 (21 ± 1 cell/1.0 × 10^−4^ mm^3^ of tissue versus 17 ± 1 cell/1.0 × 10^−4^ mm^3^ of tissue in SHAMs; *p ≤ *0.001, Cohen’s d = 1.6; Fig. 2b) and CA3 for D1 (21 ± 2 cells/1.0 × 10^−4^ mm^3^ of tissue), D3 (18 ± 2 cells/1.0 × 10^−4^ mm^3^ of tissue), and D7 (17 ± 2 cells/1.0 × 10^−4^ mm^3^ of tissue, all versus 14 ± 1 cell/1.0 × 10^−4^ mm^3^ of tissue in SHAMs; *p ≤ *0.021; Fig. 2d). Striatal neurons in the contralateral hemisphere had significantly decreased packing density in SHAM rats (19 ± 2 cells/1.0 × 10^−4^ mm^3^ of tissue) compared to ICH rats at D1 (25 ± 1 cell/1.0 × 10^−4^ mm^3^ of tissue), D3 (25 ± 2 cells/1.0 × 10^−4^ mm^3^ of tissue), and D7 (24 ± 2 cells/1.0 × 10^−4^ mm^3^ of tissue; *p ≤ *0.002; Fig. 2h). Although there was a trend towards higher cell density in S1, it did not reach statistical significance in either hemisphere (*p* ≥ 0.064; Fig. 2f). Overall, neurons were packed significantly closer together in CA3 bilaterally and contralateral striatum at all times, while CA1 cells significantly increased in packing density bilaterally on D1.

#### Hydration levels

ICH-D1, D3, and D7 rats had a higher relative percentage of abdominal muscle water content that was 2.2 ± 7.0%, 1.4 ± 6.0%, and 0.4 ± 4.7% higher compared to SHAM rats, respectively. However, these differences in muscle water content were non-significant (*p ≥ *0.703 versus SHAM for all ICH groups), so there appears to be no systemic dehydration in ICH rats.

#### FJC stain (PHZ, CA1, S1)

**Figure 3:**
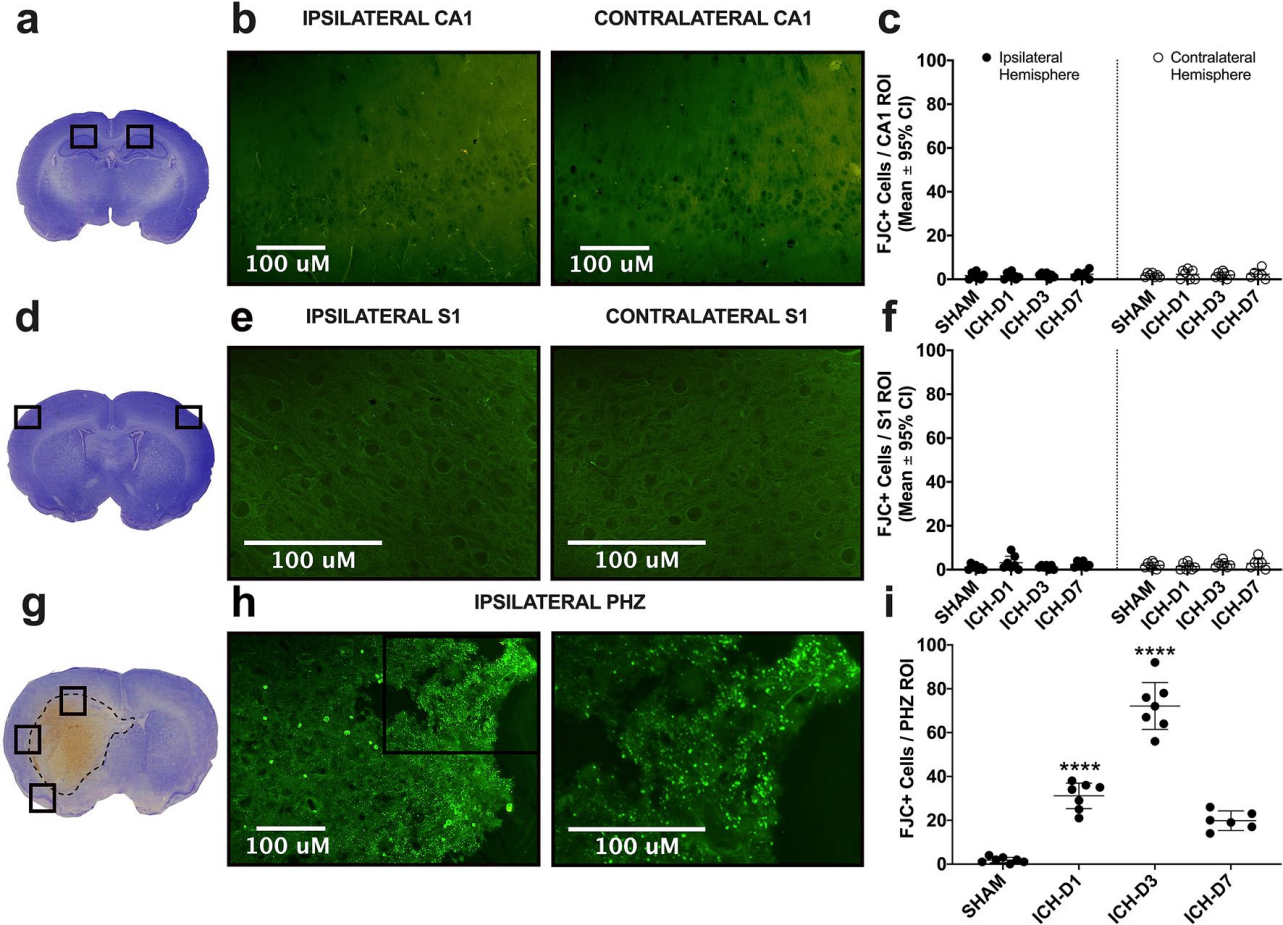
Representative sections and images demonstrating locations of regional sampling for the Fluoro-jade C positive cell counts as a marker of neuronal death in CA1, (**a**–**c**; 1.78 ± 2.89 on D1, 1.93 ± 1.15 for D3, 2.33 ± 1.97 for D7, versus 1.79 ± 1.18 for SHAMs), S1 (**d**–**f**; 3.14 ± 2.9 on D1, 1.14 ± 0.83 on D3, 2.33 ± 1.43 on D7, versus 1.5 ± 1.68 for SHAMs), and the perihematoma zone (PHZ; **g**–**i**) after ICH. A representative example of the hematoma size is delineated by the dotted black line (**g**). Representative images in CA1 were taken at 20 × objective magnification (**b**), while those in S1 were taken at 40 × (**e**). PHZ representative images (**h**) were taken at 20 × and 40 × objective magnification respectively to better demonstrate the extent of cells positive for FJC + stain, with the approximate area in which the higher magnification image was taken marked by a box on the lower magnification image. ****p < 0.0001 versus SHAM controls. Figure and scatterplots were made using GraphPad Prism version 8.4.3 for macOS, GraphPad Software, San Diego, California USA, www.graphpad.com.

There were very few FJC + cells in SHAM rats, reflecting either false positives or a low rate of natural cell death. Conversely, there were numerous FJC + cells in the perihematoma zone (PHZ; Fig. 3g–i) compared to SHAM rats at all points (*p* ≤ 0.0002). There was an effect of time on FJC counts in PHZ of the ICH rats (31.1 ± 5.84 on D1, 72.14 ± 10.7 on D3, 19.8 ± 4.5 on D7, versus 1.86 ± 1.25 for SHAMs; all *p* < 0.0001), where day 3 had the highest number of FJC positive cells and day 7 had the lowest (Cohen’s d for D1 vs. D3 = 4.4, D3 vs. D7 = 6.0, D7 vs. D1 = 2.1). Likewise, there was a comparably low number of FJC + cells in S1 (3.14 ± 2.9 on D1, 1.14 ± 0.83 on D3, 2.33 ± 1.43 on D7, versus 1.5 ± 1.68 for SHAMs) and CA1 (1.78 ± 2.89 on D1, 1.93 ± 1.15 for D3, 2.33 ± 1.97 for D7, versus 1.79 ± 1.18 for SHAMs) in all ICH groups, with no statistically significant differences (all *p ≥ *0.734 vs. SHAM; Fig. 3a–f). The PHZ serves as a positive control showing the stain’s efficacy; thus, cell shrinkage appears to be unrelated to cell death in regions far-removed from the hematoma (e.g. hippocampal layer CA1, cortical area S1).

### Experiment 2 (MCAO) results

#### Lesion volume

As expected, MCAO caused substantial cortical and subcortical injury, averaging 203 mm^3^ ± 45.2 mm^3^ (Fig. 1b). Shams had no signs of injury.

#### Cortical thickness

Rats in the MCAO-D1 group had a 0.06 ± 0.1 mm thinner cortex compared to SHAMs, a trending but non-significant effect (*p* = 0.074; Fig. 1e).

#### Contralateral cell volume (CA1, S1, striatum)

**Figure 4:**
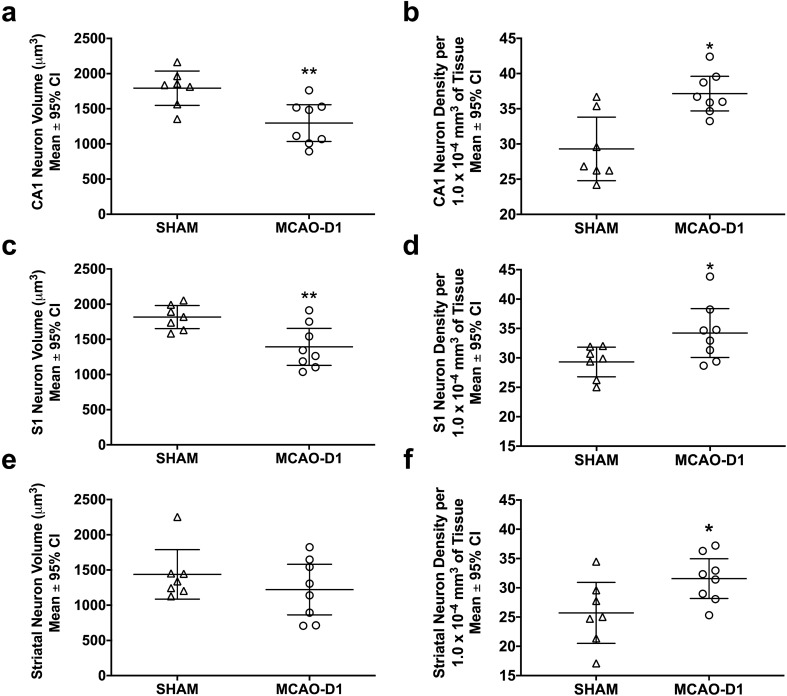
In experiment 2, contralateral neuron soma volume and density were analyzed 24 h following MCAO in CA1 (**a**, **b**), S1 (**c**, **d**), and the dorsal striatum (**e**, **f**). *p < *0.05*, **p < 0.01 versus SHAMs. Scatterplots were made using GraphPad Prism version 8.4.3 for macOS, GraphPad Software, San Diego, California USA, www.graphpad.com.

Cell volume was smaller in MCAO-D1 rats compared to SHAM rats in contralateral S1 (1393 ± 263 μm^3^ in MCAO-D1 versus 1816 ± 164 μm^3^ in SHAMs; *p* = 0.0074, Cohen’s d = 1.2; Fig. 4c) and contralateral CA1 (1297 ± 262 μm^3^ in MCAO-D1 versus 1793 ± 244 μm^3^ in SHAMs; *p* = 0.040, Cohen’s d = 1.3; Fig. 4a), but not contralateral striatum (1437 ± 351 μm^3^ in MCAO-D1 versus 1222 ± 359 μm^3^ in SHAMs; *p* = 0.321; Fig. 4e). Therefore, MCAO resulted in significant cell volume reductions in some areas at 24 h.

#### Contralateral cell density (CA1, S1, striatum)

Cell density was increased in MCAO-D1 rats in contralateral CA1 (37 ± 3 cells/1.0 × 10^−4^ mm^3^ of tissue versus 29 ± 5 cells/1.0 × 10^−4^ mm^3^ of tissue in SHAMs; *p* = 0.003, Cohen’s d = 1.4; Fig. 4b), contralateral striatum (32 ± 3 cells/1.0 × 10^−4^ mm^3^ of tissue versus 26 ± 5 cells/1.0 × 10^−4^ mm^3^ of tissue in SHAMs; *p* = 0.044, Cohen’s d = 1.1; Fig. 4f) and contralateral S1 (34 ± 4 cells/1.0 × 10^−4^ mm^3^ of tissue versus 29 ± 3 cells/1.0 × 10^−4^ mm^3^ of tissue in SHAMs; *p* = 0.034 Fig. 4d). Overall, neuronal packing density was significantly higher in CA1, S1, and striatum 24 h following ischemic stroke.

### Experiment 3 (HIE) results

The total infarct volume in the HIE group averaged 5.7 ± 1.95 mm^3^ (SM 1.2, SM Fig. 1), whereas there was no sign of injury in SHAM controls. There were no significant differences in cortical thickness or cell volume between HIE-D1 and SHAMs (*p ≥ *0.164), but there was a significantly higher contralateral CA1 cell density in the HIE-D1 group versus SHAMs (21 ± 2 cells/1.0 × 10^−4^ mm^3^ of tissue versus 24 ± 3 cells/1.0 × 10^−4^ mm^3^ of tissue in SHAMs; *p ≤ *0.021, Cohen’s d = 0.10; SM 1.1, SM Fig. 1). SM 1.1 outlines the results of Experiment 3 in more detail.

### Experiment 4 (TEM + ICH)

**Figure 5:**
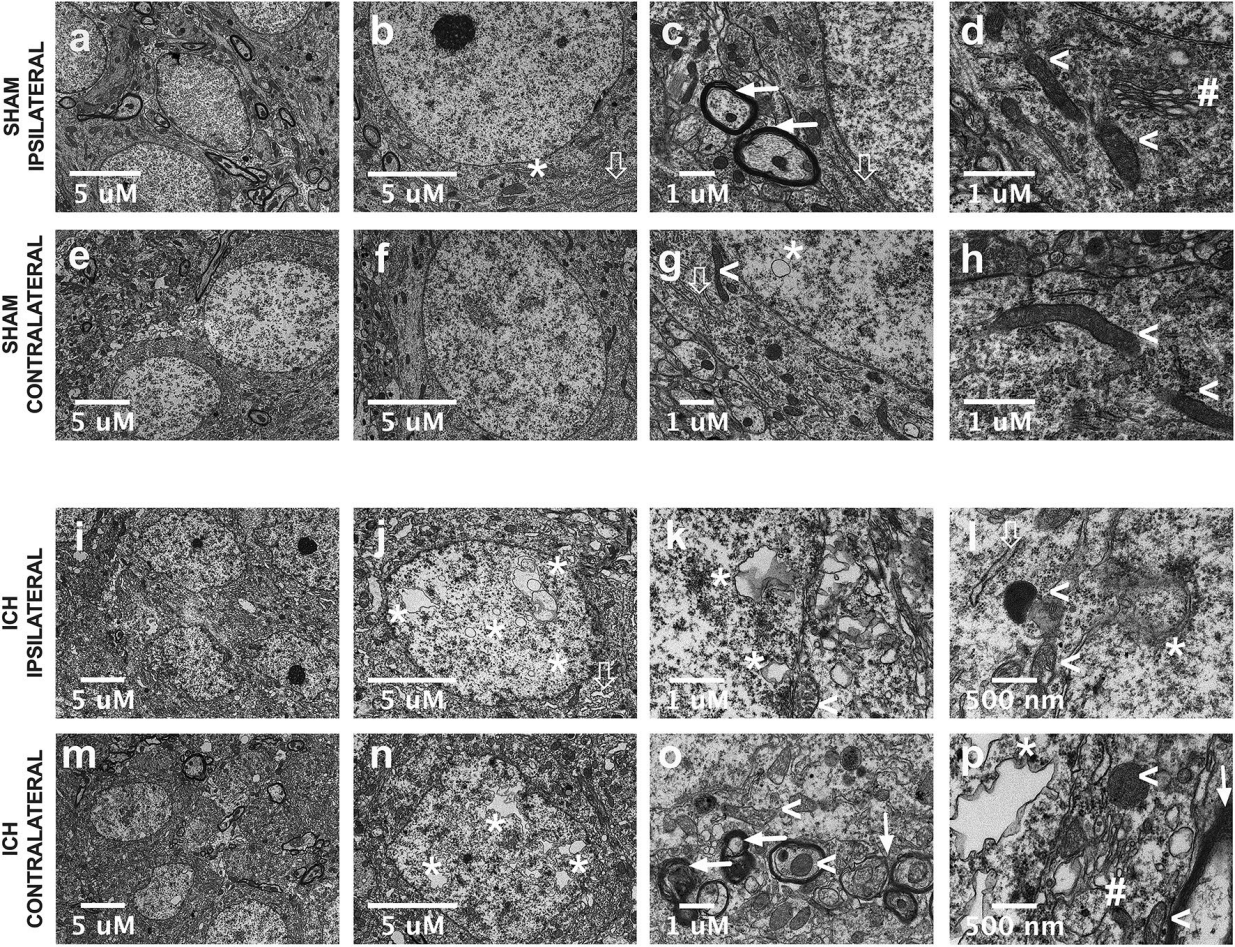
Representative transmission TEM images of hippocampal layer CA1 area in Experiment 4, in SHAM (**a**–**h**) and striatal ICH groups (**i**–**p**) at 24 h post-stroke. The asterisks identify the presence of the small intranuclear vacuoles that appear with relatively rare frequency in SHAMs. Solid arrows identify normal and intact myelinated axons, while non-solid arrows mark well-defined, narrow rough endoplasmic reticulum. Arrowhead symbols ( <) indicate intact healthy-looking mitochondria with well-defined and packed cristae, and the # symbol denotes Golgi apparatus, which looks structurally normal. In ICH rats, asterisks mark large intranuclear vacuoles that are spread throughout the nucleus, both contralaterally and ipsilaterally (**j**–**l**, **n**, **p**). Solid arrows indicate instances of axonal damage; signs of injury include thinning interrupted, unravelling, or “smudged” myelin accompanied by dilated periaxonal spaces (**o**, **p**). Mitochondria both within such axons and the cell soma are denoted by arrowhead symbols, and appeared to be extremely edematous, containing disorganized and interrupted cristae, with some in transitive stages of lysosomal degeneration (**l**, **o**, **p**). The hashtag symbol denotes a disrupted and edematous Golgi apparatus (**p**), and the non-solid arrow denotes rough endoplasmic reticulum that are swollen and distended (**j**, **l**). At low magnification, CA1 pyramidal cells in the ICH group have noticeably crenated nuclear and plasma membranes, with extremely dense cytoplasmic and nuclear contents, which we posit is the result of tissue compliance. In layer CA1, cells in the SHAM group have a characteristic ovoid shape with taught, intact membranes. Figure was made using GraphPad Prism version 8.4.3 for macOS, GraphPad Software, San Diego, California USA, www.graphpad.com.

Compared to SHAMs (Fig. 5a–h), ICH animals (Fig. 5i–p) had irregularly shaped neurons across CA1 and S1, and displayed crenated cytoplasmic and nuclear membranes (Fig. 5j, n). The majority of organelles (mitochondria, Golgi apparatus, rough endoplasmic reticulum; Fig. 5k,l,o,p) were edematous and dilated, with numerous electron dense secondary lysosomes present, many of which appeared to be mitochondrial in origin based on the presence of a double membrane and partial remnants of cristae (Fig. 5l,o,p). Notably, mitochondrial injury was observed in axonal cross-sections, accompanied by signs of both myelin and axon damage, such as dilated periaxonal spaces, axonal atrophy, thinning or interrupted myelin, and splayed lamellae (Fig. 5o,p)^29^. Many of the grey matter axons from cortex and hippocampus show signs of axonal degeneration, with secondary demyelination. However, it is unclear whether the observed axonal degeneration proceeds neuronal degeneration, or vice versa.

After ICH, there were large intranuclear vacuoles in CA1 and S1 pyramidal neurons, which occupied much of the nucleus (Fig. 5j,n). Vacuoles of similar morphology were observed in the cytoplasm, in close proximity to the nucleus (Fig. 5k). These vacuoles appear to have a double membrane, indicating that they originated from plasma membrane or nuclear envelope, making it unlikely that these observations are attributable to folds or tears caught in the plane of section. Simultaneously, we observed abnormally large nuclear envelope invaginations and evaginations coupled with active vacuole formation (i.e. those observed “pinching off” the nuclear envelope). It is not clear if this is an autophagic response, or short-term storage of re-usable plasma membrane components; occasionally, double membraned cytoplasmic vacuoles in close nuclear proximity exhibited electron dense inclusions, and some appeared to be in lysosomal transition (Fig. 5l,o,p). Such vacuoles did not display cristae or bear resemblance to mitochondria, indicating that they likely originated from nuclear or plasma membrane, given their double membrane and close proximity to actively forming nuclear vesicles. Instances of severe intranuclear vacuolation were predominantly observed in the contralesional hemisphere, but they also were observed ipsilaterally. In contrast, intranuclear vacuoles were small and rarely observed in sham tissue (Fig. 5b,g).

### Overall cell shrinkage

**Figure 6:**
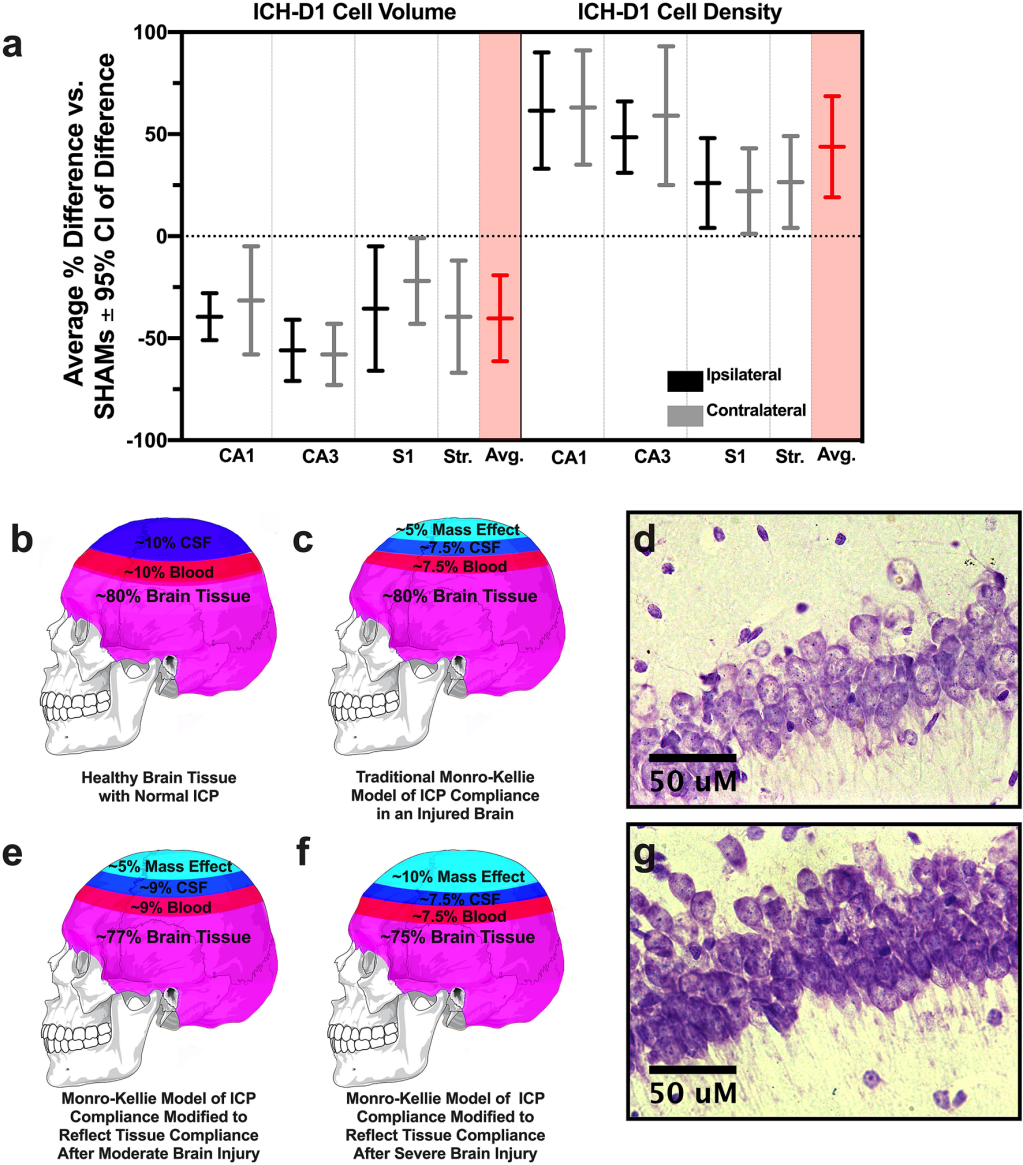
A plot of the D1 effect size (average % difference vs. SHAMs $\pm$ 95% CI of difference) for cell volume and density (**a**) across every brain region assessed in experiment 1, along with the overall experimental average effect size for each endpoint (highlighted). The traditional Munro-Kellie model of ICP compliance asserts that once a mass is added to the brain (whether that be edema and/or a hematoma), CSF and vascular blood are redirected to reduce rising ICP (**b**, **c**). In our proposed modification, the increase in neuronal packing density and reduction in cell volume that we observed in both ICH and MCAO kick in as ICP rises, aiding in compliance and reducing the required displacement of vascular blood and CSF (**e**), especially after large strokes (**f**). This may aid in avoiding secondary ischemic injury due to reduced cerebral blood flow, and potentially helps prevent transforaminal herniation. Lastly, representative cresyl violet images of hippocampal layer CA1 in both ipsilateral ICH-D1(**d**) and SHAM (**g**) tissue are shown to illustrate the tissue compliance phenomenon. Cresyl violet images were taken at 40 × magnification, and cranium diagrams (derivative of Creative Commons Licence CCO Public Domain image: https://commons.wikimedia.org/wiki/File:Human_skull_no_text_no_color.svg) were put together in Adobe Photoshop (version 21.2.1) and are not to anatomical scale. Figure was made using GraphPad Prism version 8.4.3 for macOS, GraphPad Software, San Diego, California USA, www.graphpad.com.

On D1 following ICH, the average neuronal volume reduction across regions was 42.0% (*p ≤ *0.0001, Cohen’s d = 1.8; Fig. 6a) and the average density increase was 34.0% (*p ≤ *0.0001, Cohen’s d = 0.8; Fig. 6a). This trend is similar to what we observed in astrocytes on D1, with a 41% average reduction in soma volume (Fig. 2i–j). After MCAO, cell volume reduced by an average of 22.7% (*p ≤ *0.0004, Cohen’s d = 0.2) and density increased by an average of 22.1% across regions (*p ≤ *0.007, Cohen’s d = 0.5, Fig. 4). In HIE, cell volume was 2.5% larger (*p ≥ *$0.354,$ Cohen’s d = 0.1) and there was a 14.1% increase in packing density across regions (*p ≥ *$0.050$, Cohen’s d = 0.3; SM 1.1 Fig. 1).

## Discussion

Since the inception of the Monro-Kellie doctrine, it has often been assumed that brain tissue itself plays a static role in the ICP compliance response, while CSF and blood act as the dynamic components, redirecting in attempt to maintain ICP homeostasis. However, clear in vitro evidence shows that neurons possess regulatory mechanisms to adjust cell volume in response to stressors such as high environmental pressure^11,13,30,31^; so it seems unreasonable to assume that brain tissue has no role in compliance. With careful tissue preservation, strict a priori exclusion criterion, and precise quantitative stereological assessment methods, we demonstrate that large ischemic and hemorrhagic strokes in adult rodents causes widespread tissue and cell shrinkage in areas well outside the injury. Given the nature and timing of tissue compliance, we surmise that it reflects ICP compensation following large space occupying-lesions. Notably, we found in experiment 1 that tissue shrinkage was greatest on D1 (i.e. before peak ICP at 24–72 h, historically)^16,22,23,25,26^, with a subsequent rebound in the following 6 days. We emphasize that this shrinkage is distinct from tissue deformation or midline shift, which have long been recognized. Under TEM, neurons simultaneously displayed signs of active volume regulation and sublethal injury, indicating that tissue compliance may have functional consequences throughout the brain. Overall, these findings challenge the current dogma regarding pressure–volume relationships (Fig. 6b,c), and indicates that neurons may adaptively regulate their volume in response to elevated ICP, at least after severe insults.

Following ischemic and hemorrhagic stroke, neurons across brain regions decreased in both somata volume and extracellular space (Fig. 6d,g). These data, along with our earlier findings^16^, lead us to a modified interpretation of the Monro-Kellie doctrine (Fig. 6e,f). In this revision, ICP compliance consists of the reduction of vascular blood and CSF in the cranium (well-established), along with a temporary adaptive change in tissue compliance to provide further compensation after more severe strokes. Likely, this extra compliance mechanism is only invoked following severe brain injury, as indicated by our previous serendipitous and retrospective observations in the AWB model^16^, and perhaps only after other compliance mechanisms have been heavily engaged. There is no simple way to measure the contribution of each mechanism, but consider this: the peak of the tissue shrinkage effect occurred on D1 after ICH, with a remarkable 42% average reduction in neuronal soma volume and 34% increase in packing density, along with an average soma volume reduction of 41% in astrocytes. Similarly, following MCAO, there was a 23% average reduction in contralateral neuronal soma volume, and a 22% increase in packing density at that time. Even a small reduction in cell volume and a concurrent increase in packing density, if across the brain, could free up appreciable space to buffer heightened ICP. As noted, freeing up just 5% of the volume occupied by gray matter would represent a savings of 65 mL in the human cranium, allowing considerable room for mass effect, given that the average hematoma size is ~ 35 mL^32^. High ICP is the common element of the three models where we have found tissue compliance: AWB model (mass effect predominantly due to extensive hematoma), collagenase model (mass effect due to hematoma and significant edema) and MCAO (mass effect solely from edema). Therefore, the most parsimonious explanation is that elevated ICP is primarily driving tissue compliance.

Changes in cell volume often occur through regulated processes (commonly referred to as regulatory volume decrease; RVD) in order to maintain a homeostatic set-point^33^. It is well-established that neurons and astrocytes have the capacity to reversibly regulate their volume in response to high pressure and other noxious stimuli, but the mechanosensation mechanism through which these RVDs occur has yet to be fully identified^33,34^, and is beyond this study’s scope. Recently, RVDs have been demonstrated to have mechanistic overlap with early apoptotic shrinkage^34–39^; during the first stage of apoptotic shrinkage, a neuron can decrease in volume by 20–40% via ion and osmolyte extrusion alone^35^. However, in the present study, we demonstrated via FJC^+^ cell counts that tissue compliance occurred in the absence of any notable cell death. Although RVDs and apoptotic shrinkage do share some mechanisms^35^, under TEM there were no signs of apoptosis (e.g. chromatin condensation and apoptotic bodies), supporting the FJC + count data. The fact that cell volume and cortical thickness across most regions began to rebound by D7 post-ICH without a change in cell death (vs. SHAMs) strongly suggests that the volume reduction we observed is not from apoptotic shrinkage. At the very least, it reflects the fact that these cells are not reaching the point of no return at which programmed cell death processes proceed^35^. While our experiments looking as late as D7 post-ICH suggest that shrunken cells will survive, longer term survival studies are still required to prove this.

Although we saw no definitive evidence of cell death in CA1 and S1 in Experiment 1, TEM suggests that tissue shrinkage has consequences. Despite the distance from the hematoma, there was evidence of sublethal damage (e.g. electron dense mitochondria, perturbed myelin, axonal atrophy) in neurons across both hemispheres. Compared to our past TEM work characterizing ischemic cell death^40^, it was obvious that the sub-lethal damage observed in this study did not include features of irreversible ischemic injury, which suggests that the underlying mechanisms are due to tissue compliance rather than ongoing cell death. The dramatic intranuclear/cytoplasmic vacuolization and crenated membranes indicate that neurons are actively recycling membrane to reduce cell volume, but further study is required to validate this—although we did not notice other pathological changes within the neuropil of cortex and hippocampus, it is possible that additional quantitative TEM might reveal other perturbations. Concerningly, the numerous potential signs of subcellular injury suggest that the tissue compliance processes are detrimental to cellular health, potentially impacting brain function. As a key translational step, future work should ascertain the functional impact of these histological findings.

We used multiple stroke models to determine whether or not tissue shrinkage is unique to hemorrhagic stroke. We did not intend to directly compare models, but perhaps the greater shrinkage after ICH versus ischemia is due to both the mass effect of the hematoma and edema contributing to greater elevations in ICP. However, the models were not matched for lesion size or ICP response, so future work is required to delineate those relationships. The apparent lack of tissue compliance following HIE likely reflects the developmental differences between rat pups and adults; pups do not begin to undergo skull suture fusion until PD12, which is not complete until ~ PD22^41^, although other mechanisms may be at play (e.g., greater reduction in CSF levels). Due to this developmental timeline, ICP is rarely measured in HIE studies; the cranial sutures have not yet fused by PD8, meaning that pressure is free to equalize after injury prior to suture fusion^28^. Accordingly, the tissue compliance effect following HIE appeared to negligible in experiment 3. These findings do not rule out the possibility that tissue shrinkage can occur in rat pups, as the timing or injury severity threshold to initiate tissue compliance could differ among brain injuries (e.g. later development of injury in HIE vs. adult MCAO). This also holds true for our findings in ischemic stroke; future study is required to elucidate the time course of tissue compliance in ischemia and other forms of brain injury, due to differences in ICP timing and magnitude that are dependent on the specific pathophysiology of the insult.

Two key questions are whether tissue shrinkage-mediated compliance occurs in humans, and if it is a viable therapeutic target. In order to answer those, one must consider many other issues, such as the dynamic interplay among all compliance mechanisms and ICP, as well as studying the impact of age, sex, and various co-morbidities. Shrinkage, not just of edematous tissue but distal regions too, may also be affected by osmotherapies, such as hypertonic saline or mannitol; this hypothesis is based upon the fact that regulatory volume decreases in neurons occur via the movement of ions and organic osmolytes across the plasma membrane^33,42^. While osmotherapies may augment tissue compliance, we must consider that they may also enhance subcellular perturbations, or perhaps cause cell death. There is some evidence in other clinical fields that suggests that tissue compliance may occur in humans. For example, one case study documented brain-wide cell compression as a result of tumour mass effect and high ICP in several individuals, but without global loss of neurons, similar to what we found here^43^. This at least suggests that our findings may generalize to humans. However, if tissue compliance does not occur in humans, at least acutely after stroke, it represents a fundamental species difference, and one that may help partly explain other differences between clinical stroke and animal models (e.g. higher survival rates in rodents following severe strokes). Before considering that, other factors (old age, hypertension, etc.) that may block the tissue shrinkage response have to be defined, which we are currently evaluating.

There are a number of additional considerations that remain unexplored. Astrocytes had similar volume reductions after ICH, but this was a post-hoc analysis, and further study is needed to confirm and extend those initial observations (e.g., after other forms of brain injury, in other brain regions, and with additional methods to better delineate sub-type responses). ICP was not directly measured in animals used for histology (to minimize complexity and potential confounds), however, we have repeatedly demonstrated intracranial hypertension in the same ICH and MCAO models as used presently^16,22,23,25,26^. We emphasize the challenging nature of directly relating ICP to any given compensatory mechanism due to the dynamic presence of several others, especially given the complexity of interpreting the ICP response (measured parameters could include average, peak, spiking behaviour, etc.). Due to the anatomical constraints of the cranium, simultaneously measuring ICP and other compliance components along with determining hematoma and edema volume accurately without introducing any confounds is currently impossible. Blood gases, glucose, and pressure were not measured in the present study because our past work demonstrates that these parameters only vary modestly, and are unrelated to brain injury^16,23,25,26,44–46^. More directly, in an unpublished post-hoc analysis of data from our recent study^46^, we found that fluctuations in blood pressure and glucose do not correlate with tissue compliance; we also found that anesthetic does not appear to influence tissue compliance^46^. Lastly, the studies characterizing mechanosensitive volume changes in healthy neurons have predominantly been done in vitro, and the effect of pathologically high ICP has not directly been evaluated in relation to cell volume regulatory mechanisms. As such, exploring these mechanisms in vivo in regard to distal tissue compliance remains a vital future direction if we are to more effectively and safely manipulate intracranial compliance.

Our findings challenge the long-held view that brain tissue is an immutable component of ICP pressure–volume relationship. As such, we propose a key modification to the Monro-Kellie doctrine (Fig. 6e,f) to include adaptive cell volume changes as part of a series of compliance responses to elevated ICP. We acknowledge that further work is required to determine the relative contribution of this and other mechanisms to intracranial compliance. Furthermore, the present study suggests that widespread sublethal damage occurs in neurons undergoing shrinkage, perhaps as a direct result of shrinkage mechanisms (e.g. loss of protective osmolytes), and again additional study is required to uncover the mechanism(s) responsible. If this is the case, then there is a functional cost to neurons as they reduce their volume to supplement ICP compliance, and driving it further might create additional cellular damage outside the damaged and edematous zone. Going forward, we believe that effective control of intracranial hypertension, at least for severe brain injuries, will require consideration to widespread cell volume perturbations.

## Methods and materials

### Subjects

All experiments followed the ARRIVE guidlines^47^ and the Canadian Council on Animal Care Guidelines and were approved (protocols #960, #363, and #557) by the Biological Sciences and Health Sciences Animal Care and Use Committees at the University of Alberta. In total, 82 male Sprague Dawley rats were used. We purchased 62 male Sprague Dawley rats (350–400 g, ~ 2–3 months of age) from Charles River (Saint Constant, Quebec). Long Evans rats were purchased from Charles River (Montreal, QB), bred, and delivered vaginally. Within 48 h of birth, rat litters were culled to 10 pups, reducing weight variability. The Yager Lab bred 20 male Long Evans rat pups, reared by their dams and used on post-natal days 7–8. Rats were kept in a temperature and humidity-controlled environment with a 7:00–19:00 h light cycle, during which all experiments were performed; all Sprague Dawley rats were solo-housed following surgical procedures for the duration of experiments. Water and standard rat chow were provided ad libitum*,* with water consumption monitored over the duration of experiment 1.

### Experimental protocols

In order to have confidence in these findings, we followed guidelines on translational rigour^47,48^. Our experiments used a priori power analyses to determine group sizes. We aimed to have at least 80% power to detect a 25% difference in neuron soma volume and density, based on our past tissue response work in multiple ICH models^16^. We ensured that this sample size allowed for an average stereological coefficient of error of less than 0.1 for each endpoint, as convention dictates^49–51^. The manipulation of experimental parameters such as timing and type of brain injury were used to examine the generalizability and robustness of the tissue shrinkage effect^18^. All rats were assigned to groups using a random number generator (random.org). All analyses were conducted by an experimenter blinded to group identity. Unbiased stereology was used to ensure high accuracy for all cell morphology quantification, and a strict tissue quality exclusion criterion was followed to maintain optimal conditions for stereological assessment. The experimental timelines and endpoints are summarized in Table 1.

In experiment 1 (N = 36), adult rats were given a collagenase-induced ICH, and euthanized 1 (ICH-D1; n = 8), 3 (ICH-D3; n = 8), or 7 days (ICH-D7; n = 8) later. Control rats (SHAM; n = 12) were given a sham ICH procedure, and for blinding purposes, further randomized to 1 (n = 4), 3 (n = 4), or 7-day survival times (n = 4), with the a priori intention to combine these subgroups *if no statistical differences were detected,* as there was no plausible expectation of subgroup differences. In experiment 1, neurons were assessed in areas CA1, CA3, S1, and the contralateral striatum, and astrocytes were assessed in ipsilateral CA1 and contralateral striatum. In experiment 2 (N = 16), adult rats either received an MCAO (MCAO-D1; n = 9) or sham (SHAM; n = 7) procedure and were euthanized 24 h later. Regions were only assessed contralaterally due to widespread edema and injury in the injured hemisphere. In experiment 3 (N = 20), post-natal day 7 (PD7) rat pups received either an HIE (HIE-D1; n = 10) or sham (SHAM; n = 10) procedure and were euthanized 24 h later. Areas CA1 and S1 were analyzed contralaterally in experiment 3. In experiment 4 (N = 10), adult rats received a collagenase ICH (n = 5) or a sham (n = 5) procedure, and were euthanized 24 h later. Areas CA1 and S1 were assessed bilaterally in experiment 4.

### Exclusion criteria

Exclusion criteria were determined prior to initiating the study, and applied in a blinded manner, as accurate stereological analysis relies on optimal tissue quality^50,52,53^. Prior to microscopic examination, if tissue had noticeable differences in staining penetration or other major histological processing damage (e.g. dark neurons^54^, freezing damage), rats were entirely excluded from analysis. Rats were excluded from representative regional analysis (i.e. not the overall experiment) if there were local tissue distortions in the region of interest, as these tissue disturbances can alter the local cellular density and other parameters that play a role in stereological accuracy^50,52,53^.

### Intracerebral hemorrhage

Rats were anesthetized with isoflurane (4% induction, 2% maintenance in 60% N_2_O and remainder O_2_), and maintained at 37 °C via rectal temperature probe and heating pad. The ICH surgical procedure was performed as previously described^44^. A burr hole was drilled at 0.5 mm anterior and 3.5 mm left to bregma. Bacterial collagenase (Type IV-S, Sigma, 3.0 μL of 0.6 U/μL in saline) was injected at a depth of 6.5 mm. Bupivacaine hydrochloride (0.5 mg/kg SC) was used as a post-operative analgesic and 5 mL of 0.9% saline was injected subcutaneously to ensure hydration. For sham procedures, rats were kept under isoflurane for equivalent time (~ 25 min). An incision was made, and connective tissue cleared, but no hole was drilled in order to maintain normal ICP by avoiding a drop in ICP due to equalization with atmospheric pressure, or have an increase in ICP due to an inflammatory response. Sham-operated rats had identical doses of bupivacaine and saline.

### Middle cerebral artery occlusion

Rats were anesthetized with isoflurane and maintained at 37 °C. Bupivacaine was administered subcutaneously at the ventral neck region incision site. Rats in the MCAO-D1 group were subjected to 2 h of middle cerebral artery occlusion via the intraluminal suture occlusion model, as described previously^25,55^. A 4–0 silk suture was used to temporarily ligate the right common carotid artery and the internal carotid artery. A 4–0 monofilament suture with a 0.41 mm diameter (Item #404156PK10, Doccol Corp, Redlands, CA, USA) was used to occlude the middle cerebral artery. After the procedure, the midline neck incision was closed with 4–0 vicryl absorbable suture (Ethicon, Somerville, NJ, USA), and bupivacaine was given. All rats received 5 mL of 0.9% saline subcutaneously to ensure hydration. For sham procedures, a ventral midline neck incision was made, and blood vessels were isolated but not ligated or occluded; all SHAM rats were given the same duration of anesthetic as MCAO-D1 rats, and identical doses of analgesic and saline.

### Hypoxic-ischemic encephalopathy

The classic Vannucci-Rice model of HIE was used in this experiment, as outlined previously^56^. For detailed methods, refer to Supplemental Material (SM) 1.1.

### Histology

For euthanasia, rats from experiment 1 and 3 were given 100 mg/kg of sodium pentobarbital IP, while rat pups in experiment 2 were given an isoflurane overdose; all animals were then transcardially perfused with 0.9% saline followed by 10% neutral buffered formalin (Fisher Scientific, Toronto, ON, Canada). Brains were carefully extracted, and immersed in fresh 10% neutral buffered formalin for at least a week to allow for optimal fixative penetration. Prior to cryosectioning, brains were immersed in a 30% sucrose-formalin solution until they sank, ~ 48–72 h. Beginning at a random rostral start point to ensure systematic random sampling, brains were sectioned at 20 μm, with every 10^th^ pair of serial sections kept, and placed on two slide sets. Slides were stained with cresyl violet as described previously^57^. In experiment one, an extra set of slides were taken to allow for Fluoro-Jade C staining according to the manufacturer’s protocol (#AG325, MilliporeSigma, Oaksville, ON, Canada). Tissue processing was identical across groups; however, to further address the possibility that tissue processing was somehow responsible for the tissue compliance effect, we conducted pilot work assessing cell volume and density in both transcardially perfused and immersion fixed ICH and SHAM tissue, and found no significant differences in the extent of tissue compliance between the two methods (SM 1.2).

### Hydration levels

At the time of euthanasia, abdominal muscle was taken to assess water content^58^. Please refer to SM 1.3 for detailed methods.

### Stereological analysis

Recently, it has been suggested that combining several stereological dissectors with local stereological assessment probes, such as the nucleator, may provide explanatory accuracy equivalent with serial reconstruction studies^59^. Accordingly, the following experiments performed in this study were analyzed using a combinatorial stereological approach to ensure precision and accuracy. To assess cell volume in the present study, the nucleator probe was used in combination with the physical dissector. To assess packing density, both the physical and optical dissector probes were used. For more detailed methods, please refer to SM 1.4.

### Lesion volume and cortical thickness assessment

Lesion volume and cortical thickness were assessed in experiments 1–3 using Image J software (v. 1.52A, NIH). In experiments 2–3, sections for stereology were taken so that the same tissue could be used to determine both lesion volume and cortical thickness^44^. For the lesion volume assessment, 20 μm coronal sections were assessed every 200 μm from approximately + 5.45 mm to − 6.00 mm from bregma, as done previously^44^. In experiment 1, the section containing the maximum hematoma area was assessed instead of lesion volume, as these correlate closely^60^, and we did not section through the entire lesion to accurately gauge lesion volume. In experiments 1–3, the average cortical thickness of each hemisphere was calculated by measuring the distance between the dorsal aspect of the cingulum to cortical layer II in five sections ranging + 1.89 to − 2.0 from bregma^44^.

### Transmission electron microscopy

At 24 h post-stroke, each rat in experiment four was given 100 mg/kg of sodium pentobarbital IP, and transcardially perfused with heparinized 0.9% saline followed by Karnovsky’s fixative (glutaraldehyde, paraformaldehyde, sodium cacodylate buffer, balanced to a pH of 7.4)^54^. The head of each rat was removed, placed intact into fresh fixative, and stored at 4 °C overnight to prevent histological artefact^61^. After 24 h immersed in situ, brains were removed and areas CA1 and S1 dissected out into 1 × 2 mm blocks (maintaining orientation). After processing, blocks were embedded in resin, sectioned at 90 nm, and mounted on grids. First, toluidine blue was used to stain semi-thick (1 μm) sections to locate the area of interest, then ultra-thin sections (90 nm) were taken and stained (uranyl acetate, lead citrate), then imaged via electron microscopy. Overall, ~ 2000 neurons across areas CA1 and S1 were examined. Samples were imaged under a Philips-FEI Morgagni 268 80 kV (Phillips-FEI, Oregon, USA) transmission electron microscope at various magnifications for signs of perturbation compared to SHAMs.

### Statistical analysis

Prior to analysis, the Shapiro–Wilk test was used on all datasets to assess normality; in all cases, the result was non-significant, indicating a normally distributed population. In experiments 1–3, cell volume, cell density, cortical thickness, and FJC counts were analyzed using a two-way ANOVA with Sidak’s multiple comparisons test. The two factors compared were region (within-subject) and experimental group (between-subjects). Sham rats were collapsed across time and treated as one experimental group to increase statistical power, *as there were no statistical differences due to time among sham sub-groups.* Hemisphere was originally treated as a third factor, but no hemisphere differences were found, so analysis was simplified. When main effects were significant, the simple effects found via Sidak’s test were reported. Abdominal muscle water content in experiment 1 were analyzed using a one-way ANOVA with Sidak’s test. Cohen’s d was used as a measure of effect size for all endpoints to avoid overreliance on statistical significance^62^, and was calculated using G*Power (Version 3.9.1.4). When using Cohen’s d as a measure of effect size, typical benchmark thresholds are d = 0.2, 0.5, and 0.8 for small, medium and large effects, respectively^63^. Average amount of tissue compliance across regions in each model was calculated by averaging the percent reduction in each region compared to SHAMs. Descriptive statistics are mean ± 95% confidence intervals (C.I.), as recommended^19^. All analyses were done in GraphPad Prism v.6, with the exception of experiment 1, which was analyzed using GraphPad Prism v.8. Stereology calculations were done using RStudio (Version 1.2.1335, RStudio Inc.).

## Supplementary Information

Supplementary Information 1.

Supplementary Information 2.

## Data Availability

All data is presented in a supplementary file (Supplementary Dataset 2), along with an additional supplemental material file outlining our stereology methods in further detail (Supplemental Material 1).
