# Supplementary material for: An update to the Monro–Kellie doctrine to reflect tissue compliance after severe ischemic and hemorrhagic stroke
Source: Sci Rep. 2020 Dec 16;10:22013. doi: 10.1038/s41598-020-78880-4 (PMC7745016; doi:10.1038/s41598-020-78880-4)
Supplement: Supplementary file 1 — Supplementary Information 1. [file 41598_2020_78880_MOESM1_ESM.docx]

**Title:** An Update to the Monro-Kellie Doctrine to Reflect Tissue Compliance after Severe Ischemic and Hemorrhagic Stroke

**Supplementary Material 1**

**Authors:** Anna C. J. Kalisvaart^1^, Cassandra M. Wilkinson^1^, Sherry Gu^2^, Tiffany F. C. Kung^2^, Jerome Yager^3^, Ian R. Winship^2,4^, Frank K. H. van Landeghem^2,5^, Frederick Colbourne^1,2^

**Affiliations**

1. Department of Psychology, Faculty of Science, University of Alberta, Edmonton, Alberta, Canada

2. Neuroscience and Mental Health Institute, University of Alberta, Edmonton, Canada

3. Department of Pediatrics, Faculty of Medicine and Dentistry, University of Alberta, Edmonton, Canada

4. Department of Psychiatry, Faculty of Medicine and Dentistry, University of Alberta, Edmonton, Canada

5. Department of Laboratory Medicine and Pathology, University of Alberta Hospital, Edmonton, Canada

**Corresponding Author:**

Frederick Colbourne (University of Alberta, Department of Psychology)

Email: [fcolbour@ualberta.ca](mailto:fcolbour@ualberta.ca)

**Supplementary Material 1**

**Index**

1.1- Experiment 3 (HIE) Expanded Methods and Results

1.2- Fixation/Perfusion Pilot Study

1.3- Hydration Levels (Experiment 1, ICH)

1.4- Stereological Methods and Analysis

**1.1 Experiment 3 (HIE) Expanded Methods and Results**

1.1.1 Methods

PD7 rat pups were anesthetized with isoflurane, and temperature was maintained at 37°C with a heating lamp. A midline neck incision was made to isolate the right common carotid artery, which was tied off with 5-0 silk suture. After a 2 h recovery period, pups were moved to a chamber maintained at 37°C through which humidified 8% oxygen flowed via inlet-outlet portals for 2 h to induce hypoxia-ischemia, as described previously by the Yager lab [1, 2]. Rat pups in the SHAM group were anesthetized and had their carotid arteries isolated, but not ligated, and after the appropriate anesthetic duration, they were sutured and returned to their dam.

1.1.2 Results

Lesion Volume + Cortical Thickness

The total infarct volume in the HIE group averaged 5.7 ± 1.95 mm^3^ (Fig. 1). The HIE-D1 rats had no significant difference in cortical thickness (*p=*0.13 vs. SHAM, Cohen’s d = 0.7; Fig. 1). There was an average difference in cortical thickness of 0.03 ± 0.04 mm.

Contralateral Cell Volume (S1, CA1)

There was no effect of group on cell volume in S1 (3130 ± 338 μm^3^ versus 3355 ± 430 μm^3^ in SHAMs) or CA1 (3626 ± 539 μm^3^ versus 3248 ± 214 μm^3^ in SHAMs; all *p ≥* 0.70, SM 1.1, Fig. 1).

Contralateral Cell Density (S1, CA1)

Rats in the HIE-D1 group had significantly higher cell density in contralateral CA1 (24 ± 2 cells/1.0 x 10^-4^ mm^3^ of tissue versus 20 ± 3 cells/1.0 x 10^-4^ mm^3^ of tissue in SHAMs; *p ≤* 0.02, Cohen’s d = 0.10; SM 1.1, Fig. 1). The HIE-D1 rats did not have a higher cell density in contralateral S1 (24 ± 3 cells/1.0 x 10^-4^ mm^3^ of tissue versus 21 ± 2 cells/1.0 x 10^-4^ mm^3^ of tissue in SHAMs*; p ≥* 0.073). Therefore, neuron packing density increased in CA1 after HIE.


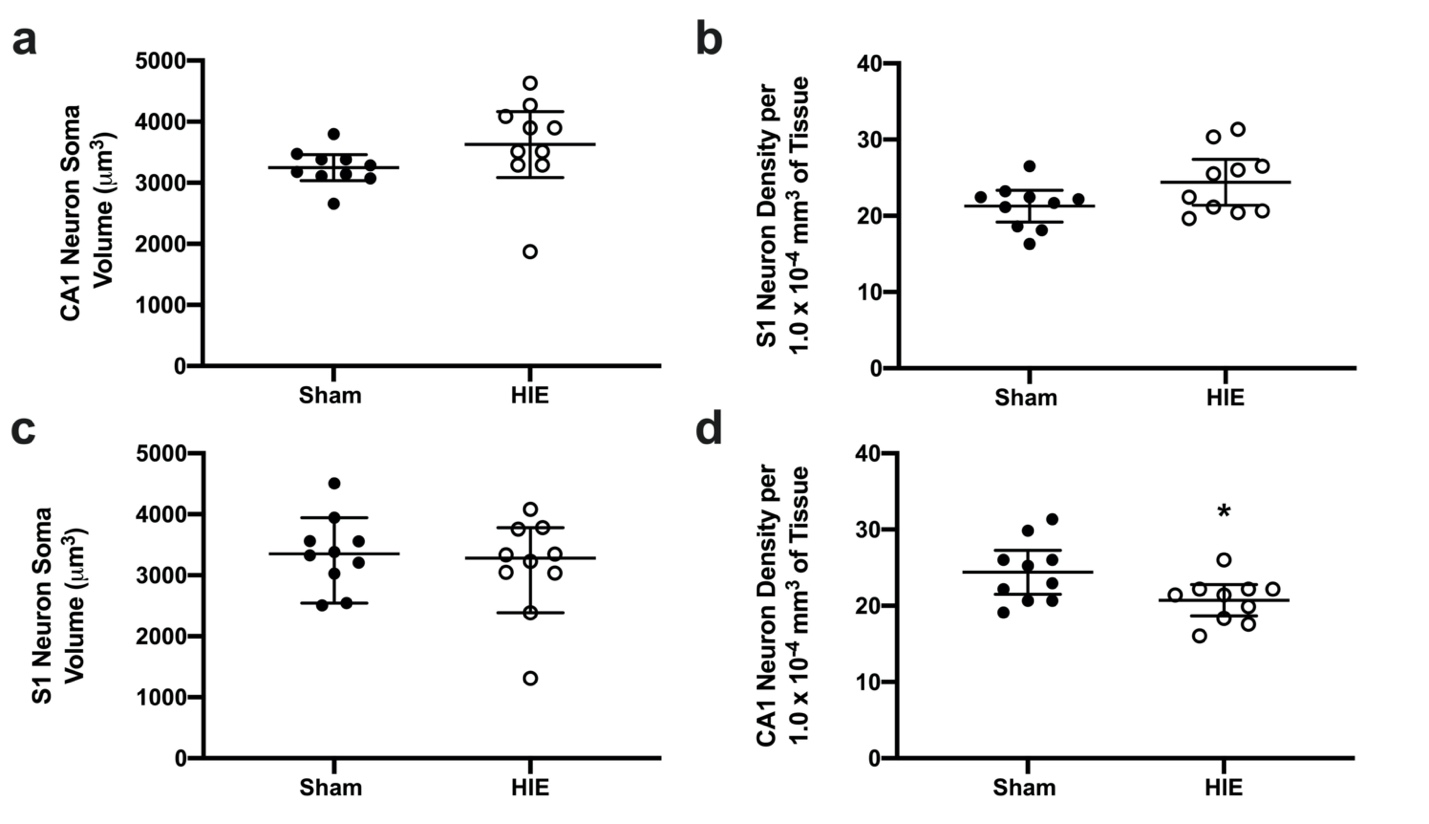


**Supplemental Fig. 1**

In experiment 3, neuron soma volume and density were analyzed 24hours post-HIE in areas CA1 (a, b) and S1 (c, d). **p* < 0.05 vs SHAM.

**1.2 Fixation/Perfusion Pilot Study**

1.2.1 Methods

Eight male Sprague-Dawley rats (350-400g) were purchased from Charles River (Saint Constant, Quebec) and randomized into two groups (Perfused; n=4 vs. Post-Fixed; n=4). All rats were given a collagenase-induced intracerebral hemorrhage according to the procedure described in the main text and euthanized 24 h post-ICH. Both groups were administered a lethal dose of sodium pentobarbital, and once under the plane of anesthetic, rats were either transcardially perfused with saline followed by formalin (Perfused), or immediately decapitated and immersion-fixed (Post-fixed). The brain tissue from both groups were left at least 7 days immersed in formalin, followed by immersion in 30% sucrose-formalin solution until the tissue sank to the bottom of the jar (~48-72 h). The brain tissue from both groups was then cryosectioned and stained as outlined in the main text. Stereological analysis was carried out as detailed below (SM 1.4). Data were analyzed via two-tailed Welch’s t-test.

1.2.2 Results

There were no significant differences in either CA1 volume (p $\geq$0.26) or density (p $\geq$0.43)

between the two groups (Perfused vs. Post-Fixed; SM 1.2 Fig. 2).

**
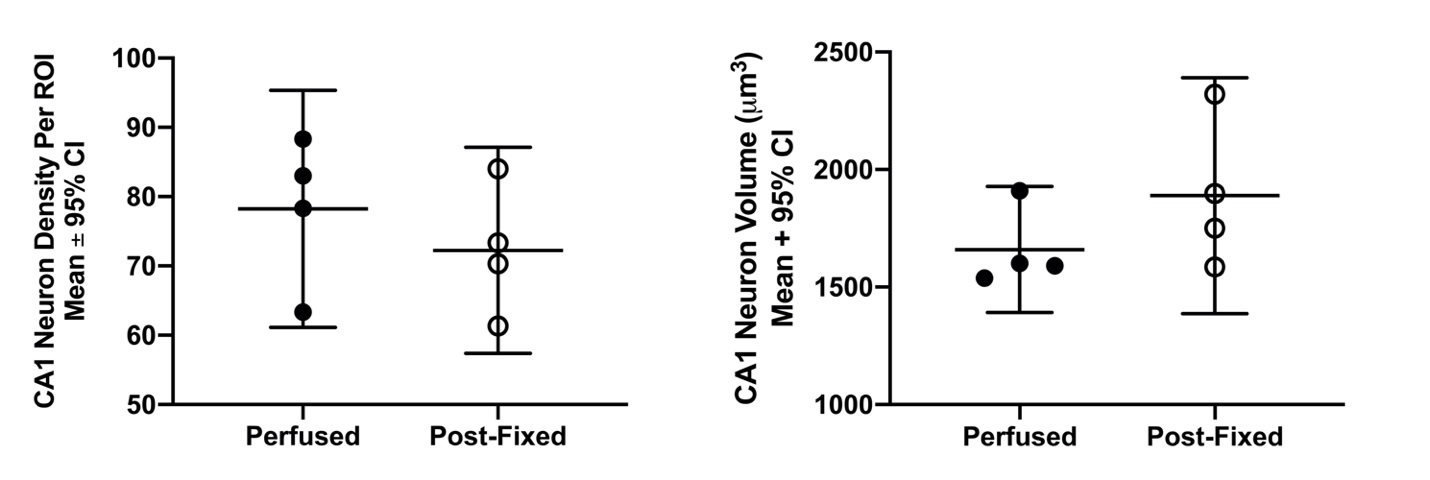
**

**Supplemental Fig. 2**

Scatterplots of CA1 neuron density and volume in perfused vs. post-fixed rats 24 h after intracerebral hemorrhage.

**1.3 Hydration Levels (Experiment 1, ICH)**

1.3.1 Methods

At the time of euthanasia, abdominal muscle was taken to assess water content, as done previously [3]. Wet weight was noted, and samples were dried in an oven for 24 h at 100°C. Dry weight was then recorded, and total water content was expressed as a weight corrected percentage: [(wet weight – dry weight)/wet weight] $\times$ 100.

1.3.2 Results

ICH-D1, D3, and D7 rats had a higher relative percentage of abdominal muscle water content that was 2.2 ± 7.0%, 1.4 ± 6.0 %, and 0.4 ± 4.7% higher compared to SHAM rats, respectively. However, these differences were non-significant (*p ≥* 0.70 vs. SHAM), so there was no evidence of systemic dehydration in ICH rats (SM 1.3, Fig. 3)


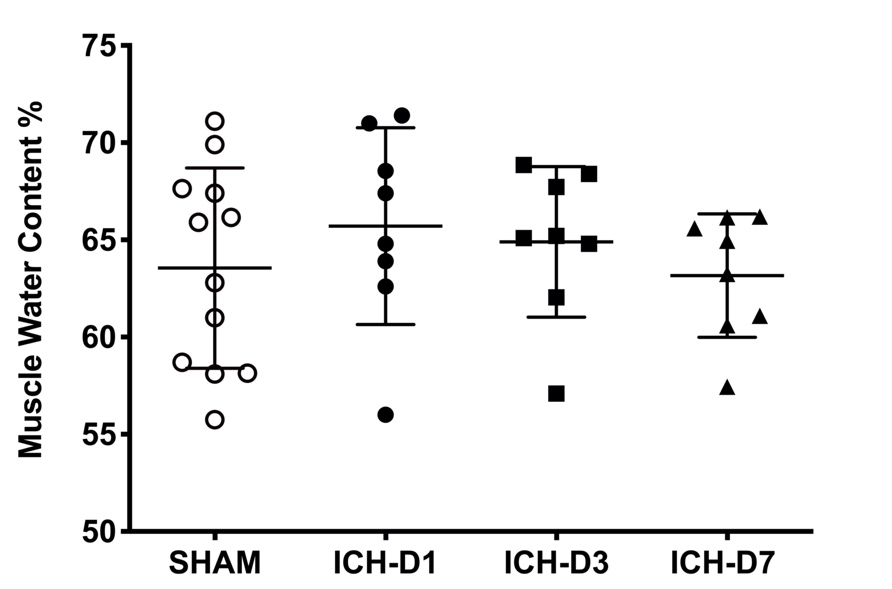


**Supplemental Fig. 3**

A scatterplot of weight-corrected abdominal muscle water content expressed as a percentage.

**1.4 Stereological Methods and Analysis**

1. Stereological Probes

Neuronal and astrocyte soma volume in each region of interest was quantified using the stereological physical dissector to aid in particle selection while using the nucleator probe [4, 5]. Neuronal numerical density was quantified using a combination of the physical fractionator and the optical fractionator [5–8]. The following methods are a cost-effective way to implement stereological analysis without specialized equipment and software, and although it is time consuming, it can be done with relative efficiency when coupled with an appropriately powered experimental design. Additionally, it has recently been suggested that combining stereological probes such as the physical fractionator and the optical fractionator with local stereological assessment probes such as the nucleator, may aid providing a greater explanatory accuracy equivalent with serial reconstruction studies [9], which is why experiments performed in this study were analyzed using this combinatorial stereological approach.

The physical dissector requires use of thin sections that are approximately a third of a height of the particle in question. However, the soma of pyramidal and glial cells can vary widely in size, ranging from 10-120 μm in diameter depending on the region in question [10]. It is not practical to section at different widths depending on the average soma size within the area of interest, and this would invalidate the systematic random sampling scheme required for these stereological probes. Therefore, cryostat sections with a nominal thickness of 20 μm were used in order to accommodate assessment of neurons smaller than the section width by the optical fractionator, but serial pairs of sections to form physical dissectors were also used to aid in both particle selection and exclusion.

1. Sampling Scheme

To determine the sampling area, the region of interest was delineated using a rat stereotaxic atlas [11]. Within this sampling area, at 200× magnification, samples were randomly selected by overlaying a 5 mm × 5 mm reticular eyepiece grid with 1 mm × 1 mm dimensions. The approximate xy distance of the sample from a distinct anatomical landmark was measured in order to reproduce the same sample in each section of the dissector pair. In each pair of dissectors, the selected sample was photographed at 400× magnification using a camera-equipped Olympus BX51 upright light microscope. The exception to this procedure was experiment three, in which each selected sample was photographed at 1000$\times$ magnification under oil immersion due to the high density of neurons in PD8 rat pups [12].Therefore, the sampling frame in experiments 1-2 had dimensions of 225 μm × 160 μm (an area of 36,000 μm^2^), and the sampling frame in experiment 3 had dimensions of 140 μm × 100 μm (an area of 14,000 μm^2^). A stereological counting frame grid, with each frame measuring 465 μm^2^, was randomly superimposed over the sampling frame photographs using ImageJ (v. 1.52A, NIH). resulting in approximately 77 sub-sample counting frames in each sample. This method has been shown to have higher precision in estimating neuronal number because the analysis of neuron number using numerous individual subsampling frames are less variable than estimates based on a smaller number of large frames [13].

1. Numerical Density

Both the physical dissector and the optical fractionator were used to assess the numerical density of neuronal cells in each representative area [6, 13, 14]. Similar to others without access to automated z-axis measurement [15], approximate dissector height was determined by focusing through the depth of field of an object of known thickness, and denoting gradations of the microscope fine adjustment knob. Average section thickness after processing was found to be approximately 10.5 μm, which aligns with the 40-70% post-processing shrinkage for cryostat sections found by others [16–19]. Thick frozen sections are not typically vulnerable to major differential z-axis collapse, so use of guard zones of the same width on either side of the plane of focus is acceptable [17]. The fixed height of the optical dissector must be less than the thickness of the section to avoid counting error. These errors can result due to the introduction of artifact (such as ‘lost caps’ and distortion of the z-axis) at each cutting surface, which is the purpose of the guard zones [19]. Guard zones of approximately 2 μm were used at each cutting surface of the section, leaving an optical dissector with a height of 6.5 μm.

Neurons were counted by according to stereological rules for the unbiased counting frame based on whether the top of their nucleus was present [4–6]. They were distinguished from other cell types by using the excellent morphology algorithm developed by Garcia-Cabezas et al. 2016 [16], which differentiates neurons and glia based on presence of a nucleolus, Nissl bodies, and distinct heterochromatin distribution patterns. To account for the possibility that the soma of some neurons could be larger than the thickness of the section, and therefore be present in multiple sections, physical dissector pairs were used to check whether any neurons counted in the reference section were also present in the look-up section, in which case they were disqualified from the count. This ensures that only unique points in the sampling frames were counted. Physical dissector pairs were separated by constant distance of 80 μm.

The volume of the sampling fraction was determined as previously recommended [9], by multiplying the length, width, and height of the sampling frame (225 × 160 × 10.5), giving a volume of 378,000 μm^3^. This was converted to cubic millimeters, providing a volume fraction of 3.78 × 10^-4^ mm^3^ per sampling frame. Numerical density data was expressed as proportion of total points counted per a volume fraction of 1.0 × 10^-4^ mm^3^ in each animal per area.

1. Soma Volume

Neuron and astrocyte soma volume was determined by use of the nucleator probe and physical dissector [4, 5, 22]. Photographs of the sampling frame in the reference and look-up section used for numeric density were also used to determine soma volume. A grid with an area per square of 75 μm was overlaid over the sampling frame images with a random offset. If a given neuron’s nucleolus intersected a grid point, it was selected for volume quantification. Astrocytes were also assessed using the nucleator point, but because the nucleolus was not visible (as is often the case), a central mark was drawn on each astrocyte before superimposing the selection grid instead [22]. Five neurons or astrocytes per sampling frame were assessed across all sampling frames.

Once a neuron was randomly selected for analysis, four rays were draw using ImageJ’s line tool from the intersection of the grid point in the nucleolus or central mark to the edge of the soma, ensuring consistency. The length was measured in micrometers, then cubed, and the mean of the cubed lengths was used to calculate an estimation of soma volume [4]:

$$\boldsymbol{\nu}_{\boldsymbol{N}}\boldsymbol{=}\frac{\boldsymbol{4}\boldsymbol{\pi}}{\boldsymbol{3}}\boldsymbol{l}_{\boldsymbol{n}}^{\boldsymbol{3}}$$

*Where:*

*V_N_ is the mean volume estimate of particles sampled*

*L^3^_n_ is the mean of the cube of the length of rays*

1. Unbiased Counting Frame Grid

In experiment 1, an unbiased two-dimensional counting frame grid was superimposed with random offset on FJC images taken at 40× magnification in each area of interest [20]. Stereological counting rules were used to determine the average number of FJC positive cells in each region.

1. Coefficients of Error

The estimated coefficient of error values provided in this study were calculated using the Gundersen methods [4, 5, 7, 21]. To calculate the coefficient of error of the neuronal volume estimation, the average area/volume estimate across each representative brain region in every animal was first determined using the following formula:

$$\boldsymbol{R}\boldsymbol{=}\frac{\boldsymbol{1}}{\boldsymbol{n}} \sum_{\boldsymbol{i=1}}^{\boldsymbol{n}} \boldsymbol{Ri}$$

*Where:*

*n = Number of nucleator estimates*

*Ri = Area/volume estimate for each sampling site*

Then, the estimated coefficient of variation for neuronal volume approximations was determined using the following formula:

$$\boldsymbol{CV}\left( \boldsymbol{R} \right)\boldsymbol{=}\frac{\sqrt{\frac{\boldsymbol{1}}{\boldsymbol{n-1}}\sum\begin{aligned} \boldsymbol{n} \\ \boldsymbol{i=1} \end{aligned}{\boldsymbol{(Ri-}\bar{\boldsymbol{R}}\boldsymbol{)}}^{\boldsymbol{2}}}}{\bar{\boldsymbol{R}}}$$

*Where:*

*n = Number of nucleator estimates*

$\bar{R}$ *= Average area per volume estimate for each sampling site*

*Ri = Area/volume estimate for each sampling site*

Then, using the estimated coefficient of variation, the coefficient of error for neuronal volume approximations was determined using the following formula:

$$\boldsymbol{CE}\left( \boldsymbol{R} \right)\boldsymbol{=}\frac{\boldsymbol{CV(R)}}{\sqrt{\boldsymbol{n}}}$$

*Where:*

*CV(R) = Estimated coefficient of variation*

*n = Number of nucleator estimates*

To calculate the coefficient of error of the numerical density approximation, the variance due to systematic random sampling was first determined using the following formula:

$$\boldsymbol{VAR}_{\boldsymbol{SRS}}\boldsymbol{=}\frac{\boldsymbol{3}\left( \boldsymbol{A-}\boldsymbol{s}^{\boldsymbol{2}} \right)\boldsymbol{-4}\boldsymbol{B+C}}{\boldsymbol{240}}\boldsymbol{, m=1}$$

*Where:*

$$\boldsymbol{A=}\sum_{\boldsymbol{i=1}}^{\boldsymbol{n}} \left( \boldsymbol{Q}_{\boldsymbol{i}}^{\boldsymbol{-}} \right)^{\boldsymbol{2}}$$

$$\boldsymbol{B=}\sum_{\boldsymbol{i=1}}^{\boldsymbol{n-1}} \boldsymbol{Q}_{\boldsymbol{i}}^{\boldsymbol{-}}\boldsymbol{*}\boldsymbol{Q}_{\boldsymbol{i+1}}^{\boldsymbol{-}}$$

$\boldsymbol{C=}\sum_{\boldsymbol{i=1}}^{\boldsymbol{n-2}} \boldsymbol{Q}_{\boldsymbol{i}}^{\boldsymbol{-}}\boldsymbol{*}\boldsymbol{Q}_{\boldsymbol{i+2}}^{\boldsymbol{-}}$

*s^2^ = Total Particles counted (Variance due to noise)*

Then, the variance due to systematic random sampling was used to calculate the final coefficient of error for numerical density:

$$\boldsymbol{CE=}\frac{\sqrt{\boldsymbol{Total Variance}}}{\boldsymbol{s}^{\boldsymbol{2}}}$$

*Where:*

*Total Variance = S^2^ + VAR_SRS_*

*S^2^ = Total Particles counted (Variance due to noise)*

References

1. Askalan R, Gabarin N, Armstrong EA, et al (2015) Mechanisms of neurodegeneration after severe hypoxic-ischemic injury in the neonatal rat brain. Brain Res 1629:94–103

2. Nguyen A, Armstrong EA, Yager JY (2015) Unilateral Common Carotid Artery Ligation as a Model of Perinatal Asphyxia: The Original Rice–Vannucci Model. Neuromethods 104:1–13

3. MacLellan CL, Davies LM, Fingas MS, Colbourne F (2006) The Influence of Hypothermia on Outcome After Intracerebral Hemorrhage in Rats. Stroke 37:1266–1270

4. Gundersen HJG (1988) The nucleator. J Microsc 151:3–21

5. Møller A, Strange P, Gundersen HJG (1990) Efficient estimation of cell volume and number using the nucleator and the disector. J Microsc 159:61–71

6. West MJ, Gundersen HJG (1990) Unbiased stereological estimation of the number of neurons in the human hippocampus. J Comp Neurol 296:1–22

7. Gundersen HJG, Bagger P, Bendtsen TF, et al (1988) The new stereological tools: Disector, fractionator, nucleator and point sampled intercepts and their use in pathological research and diagnosis. APMIS 96:857–881

8. Gundersen HJG, Jensen EB (1987) The efficiency of systematic sampling in stereology and its prediction*. J Microsc 147:229–263

9. Napper RMA (2018) Total Number is Important: Using the Disector Method in Design-Based Stereology to Understand the Structure of the Rodent Brain. Front Neuroanat 12:16

10. Johns P (2014) Neurons and glial cells. In: Clinical Neuroscience. Churchill Livingstone, pp 61–69

11. Paxinos G, Watson C (1998) The rat brain in stereotaxic coordinates, 4th ed. Academic Press, San Diego

12. Smith AM, Pappalardo D, Chen W-JA (2008) Estimation of neuronal numbers in rat hippocampus following neonatal amphetamine exposure: A stereology study. Neurotoxicol Teratol 30:495–502

13. Schmitz C, Korr H, Perl DP, Hof PR (2001) Advanced use of 3-D methods for counting neurons. Trends Neurosci 24:377–80

14. Delaloye S, Kraftsik R, Kuntzer T, Barakat-Walter I (2009) Does the physical disector method provide an accurate estimation of sensory neuron number in rat dorsal root ganglia? J Neurosci Methods 176:290–297

15. Korkmaz A, Tümkaya L (1997) Estimation of the section thickness and optical disector height with a simple calibration method. J Microsc 187:104–109

16. Brown DL (2017) Practical Stereology Applications for the Pathologist. Vet Pathol 54:358–368

17. Gardella D, Hatton WJ, Rind HB, et al (2003) Differential tissue shrinkage and compression in the z-axis: implications for optical disector counting in vibratome-, plastic-and cryosections. J Neurosci Meth 124:45–59

18. Deniz ÖG, Altun G, Kaplan AA, et al (2018) A concise review of optical, physical, and isotropic fractionator techniques in neuroscience studies, including recent developments. J Neurosci Methods 310:45–53

19. Boyce RW, Dorph-Petersen K-A, Lyck L, Gundersen HJG (2010) Design-based stereology: introduction to basic concepts and practical approaches for estimation of cell number. Toxicol Pathol 38:1011–25

20. Gundersen HJG, Bagger P, Bendtsen TF, et al (1988) The new stereological tools: Disector, fractionator, nucleator and point sampled intercepts and their use in pathological research and diagnosis. APMIS 96:857–881

21. Gundersen HJG, Jensen EB V., Kieu K, Nielsen J (1999) The efficiency of systematic sampling in stereology - reconsidered. J Microsc 193:199–211

22. Lee, T.T., Skafidas, E., Dottori, M. *et al.* (2017) No preliminary evidence of differences in astrocyte density within the white matter of the dorsolateral prefrontal cortex in autism.*Molecular Autism* 8, 64.
